# Retinal capillary involvement in early post-COVID-19 patients: a healthy controlled study

**DOI:** 10.1007/s00417-020-05070-3

**Published:** 2021-02-01

**Authors:** Maria Cristina Savastano, Gloria Gambini, Grazia Maria Cozzupoli, Emanuele Crincoli, Alfonso Savastano, Umberto De Vico, Carola Culiersi, Benedetto Falsini, Francesco Martelli, Angelo Maria Minnella, Francesco Landi, Francesco Cosimo Pagano, Stanislao Rizzo, Gemelli Against COVID-19 Post-Acute Care Study Group

**Affiliations:** 1 Ophthalmology Unit, “Fondazione Policlinico Universitario A. Gemelli IRCCS”, Rome, Italy; 2 Catholic University of “Sacro Cuore”, Largo A. Gemelli 8, 00198 Rome, Italy; 3 Department of Cardiovascular and Endocrine-Metabolic Diseases, and Ageing, “Istituto Superiore di Sanità”, Rome, Italy; 4 Department of Geriatrics, Neurosciences and Orthopedics, “Fondazione Policlinico Universitario A. Gemelli IRCCS”, Rome, Italy; 5 “Consiglio Nazionale delle Ricerche, Istituto di Neuroscienze”, Pisa, Italy

**Keywords:** COVID-19, OCT angiography, Retinal vascular layers, Macula, SARS-CoV-2

## Abstract

**Background:**

Systemic vascular involvement in COVID-19 has been identified in several patients: not only endothelial derangement and increased permeability are reported to be early hallmarks of organ damage in patients with COVID-19 but are also the most important cause of worsening of clinical conditions in severe cases of SARS-CoV-2 infection. There are several reasons to hypothesize that the eye, and the retina in particular, could be a target of organ damage in SARS-CoV-2 infection.

**Methods:**

This cohort observational study analyzes OCT angiography and structural OCT of 70 post-COVID-19 patients evaluated at 1-month hospital discharge and 22 healthy control subjects. Primary outcomes were macular vessel density (VD) and vessel perfusion (VP); structural OCT features were evaluated as secondary outcomes. In addition, patients and healthy volunteers were evaluated for best corrected visual acuity, slit lamp photograph, and fundus photo image.

**Results:**

VD and VP in 3 × 3 and 6 × 6 mm scans for SCP and DCP showed no significant differences between the groups. Similarly, CMT and GCL did not reveal significant differences between post-COVID-19 and healthy patients. Nine patients (12.9%) featured retinal cotton wool spots and 10 patients had vitreous fibrillary degeneration. The prevalence of epiretinal membrane and macular hole was similar in the two groups. One case of extra papillary focal retinal hemorrhage was reported in the post-COVID-19 group.

**Conclusions:**

Macula and perimacular vessel density and perfusion resulted unaltered in mild post-COVID-19 patients at 1-month hospital discharge, suggesting no or minimal retinal vascular involvement by SARS-CoV-2.

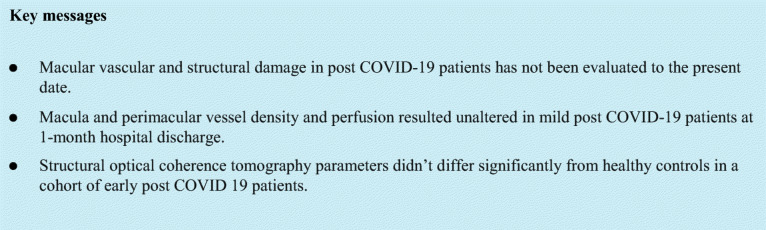



## Introduction

COVID-19 epidemic started in Wuhan province in December 2019 and rapidly evolved into a severe pandemic [1]. The disease is caused by the newly discovered SARS-CoV-2 which has been proven to determine multi-organ impairment, mostly due to innate immune response overactivation [2]. Respiratory tract involvement is the major clinical manifestation of the infection: in the most severe cases, lungs show exudative diffuse alveolar damage with massive capillary congestion often accompanied by microthrombi despite anticoagulation [3].

Systemic vascular involvement in COVID-19 has been identified in several patients: not only endothelial derangement and increased permeability are reported to be early hallmarks of organ damage in patients with COVID-19 [4] but are also the most important cause of worsening of clinical conditions in severe cases of SARS-CoV-2 infection [5]. There are several reasons to hypothesize that the eye, and the retina in particular, could be a target of organ damage in SARS-CoV-2 infection. First of all, angiotensin-converting enzyme 2 (ACE2), which was found to be one of the entry sites of SARS-CoV-2 within the human capillaries and venules pericytes [6], has also been found in the eyes in connection with Muller cells, RPE, and pericytes of endothelial cells providing a critical role in retinal neurovascular function [7]. In addition, recent findings detected SARS-CoV-2 viral RNA in the retina of 3 out of 14 deceased COVID-19 patients [8]. Lastly, coronaviruses can cause pyogranulomatous anterior uveitis, choroiditis with retinal detachment, retinal vasculitis, and virus-induced retinal degeneration in feline and murine species.

The human eye allows direct optical access to the retina and its vasculature using non-invasive optical techniques. In recent years, the development of optical coherence tomography angiography (OCTA) has changed the approach of clinicians and scientists to retinal vascular analysis [9]. OCTA is able to separately visualize superficial and deep macular capillary plexa and to provide precise structural measurements from the largest retinal vessels down to the capillaries [10].

The aim of the study is therefore to detect a macular microvascular impairment in early post SARS-CoV2 patients comparing them to the general population using OCTA imaging.

## Methods

### Study design and patients’ selection

This observational retrospective institutional cohort study was supported by Fondazione Policlinico A. Gemelli IRCSS, Catholic University of “Sacro Cuore” Rome, Italy, and designed by the investigators of Gemelli Against COVID Post-Acute Care Study Group [11]. Patients who were admitted to hospital from 1st March 2020 to 1st June 2020 due to SARS-CoV2 infection and subsequently recovered from the disease were randomly selected from the hospital databases to take part of the COVID-19 study group. Inclusion criteria to this group were infection testified by 2 successive oropharyngeal swabs positive for SARS-CoV-2 genome, healing from the disease (proven by 2 consecutive negative swabs, resolution of symptoms, serum detection of anti-SARS-CoV-2 IgGs) and at least one month interval from hospital discharge.

A control group of healthy patients was randomly chosen from hospital patients. Inclusion criteria were 2 successive oropharyngeal swabs negative for SARS-CoV-2 genome and absence of symptoms suggestive of SARS-CoV-2 infection during the previous months. Exclusion criteria for both groups were high myopia (≥ 6 diopters) [12, 13], choroidal atrophy, previously diagnosed glaucoma, retinal occlusive diseases, choroidal neovascularization, central serous chorioretinopathy, infectious choroiditis, ongoing chemotherapy [14], and drug abuse.

The study was approved by the Catholic University/Fondazione Policlinico A. Gemelli IRCCS Institutional Ethics Committee (protocol ID number: 003220/20). For each patient, informed consent was collected and a complete explanation of the target protocol was fully provided, in conformity to the declaration of Helsinki. All the authors reviewed the manuscript and vouch for the accuracy and completeness of the data and for the adherence of the study to the protocol.

### Procedures and instruments

All patients underwent a complete ophthalmological examination which included best corrected Snellen visual acuity (BCVA), slit lamp photograph (SL9900 Slit Lamp, CSO, Florence, Italy), fundus photo image (Cobra HD Fundus Camera, CSO, Florence, Italy), OCT and OCTA analysis (Zeiss Cirrus 5000-HD-OCT Angioplex, sw version 10.0, Carl Zeiss, Meditec, Inc., Dublin, USA). The right eye was randomly chosen for the assessment.

OCT acquired scans were high resolution 5 line HD scan at posterior pole and macular cube (200 × 200). The subfoveal choroidal thickness (SCT) was manually measured on cross-sectional OCT B-scans [15]. Two independent masked graders individually assessed all choroidal thickness measurement in the fovea region, from the rear edge of the RPE to the choroid-sclera junction. OCT-A imaging was performed using a 3 × 3 mm or a 6 × 6 mm volume scan pattern centered on the fovea. Zeiss Cirrus 5000-HD-OCT Angioplex has a scan rate of 68,000 A-scans per second, central wavelength of 840 nm, motion tracking to reduce motion artifact, and uses an optical microangiography (OMAG) algorithm for analysis [16]. An image of the superficial capillary plexus (SCP) and deep capillary plexus (DCP) was generated using automated layer segmentation, corrected by manual readjustments of the segmentation lines. Image processing was performed using MATLAB v7.10 (Mathworks, Inc.). VD was expressed in percentage derived from the ratio of the total vessel area (all white pixels, defined as pixels with a ratio value between 0.7 and 1.0) to the total area of analyzed region (size of the image in pixels). Angioplex software quantified the average VP using a grid overlay according to the standard ETDRS subfields. VP was defined as the total area of perfused retinal microvasculature per unit area in a region of measurement. FAZ perimeter was calculated as the length of the contour based on pixel-to-pixel distance in a scale and was expressed in millimeters. The area of FAZ was measured by counting the total number of pixels within FAZ in a scale multiplying the dimension of a pixel and expressed in square millimeters [17].

### Outcome measures and confounders

The main outcomes were differences in SCP and DCP vessel density and vessel perfusion between the two study groups. Differences in FAZ area and perimeter, subfoveal choroidal thickness, central foveal thickness, ganglion cell complex average thickness, and RNFL average thickness were considered as secondary outcomes.

The following were analyzed as potential confounders: presence of vitreomacular traction [18], epiretinal membrane(H. [19]), macular hole [20], myopia [21], previous vitreoretinal surgery [22], diabetes [23], systemic arterial hypertension [24], cognitive impairment [25], previous stroke [26], chronic kidney disease [27].

### Statistical analysis

The sample size calculation was performed using G*power (3.1.9.7 software) by setting the desired power of the study to 80%, the alpha error to 5%, and a clinically significant difference of 5% in VD. Statistical analysis was conducted using SPSS software (IBM SPSS Statistics 26.0). As concerns quantitative variables, normality of the distribution was evaluated using Lilliefors corrected Kolmogorov-Smirnov test and univariate comparison between the 2 groups was performed using a 2-tailed *T* test for independent groups. Linear correlations were established using Spearman’s test. Qualitative variables were confronted by means of a chi^2^ test or Fisher exact test when appropriate. The Bonferroni post hoc correction was applied in case of multiple comparisons. The agreement between the two graders in manual measurements (subfoveal choroidal thickness and FAZ perimeter) was determined through intraclass correlation coefficient.

Logistic regression analysis was performed to evaluate the actual strength of the associations detected by the univariate analysis. A *p* value < 0.05 was considered as statistically significant.

## Results

A total of 70 post-COVID-19 patients with a mean age of 53.7 ± 14 years (39 males, 31 females) were evaluated at one month after discharge. A control group of 22 healthy patients (8 males, 14 females) was assessed for comparison. Healthy patients’ mean age was 44.7 ± 11 years, significantly younger than that of post-COVID-19 patients (*p* = 0.006). The post-COVID-19 group was characterized by a mean of 60.3 ± 13.6 days from the onset of symptoms and 36.1 ± 12.9 days from hospital discharge. Supportive therapy included oxygen therapy in 45.8%, NIV in 15.7%, and mechanical ventilation in 5.7% of the patients; 12.9% of the participants from the post-COVID-19 group spent part of their hospital stay in intensive care unit. Lastly, most of the patients (77.3%) were treated with hydroxychloroquine during hospital admission.

Patients in the post-COVID-19 group showed a significantly higher prevalence of systemic arterial hypertension (*p* = 0.047) and diabetes (*p* = 0.003). In addition, mean BMI in post-COVID-19 patients was higher than that of patients in the control group and displayed a trend to linear correlation with SCP perfusion in 6 × 6 mm acquisitions (*R* = − 0.2, *p* = 0.071) which was not present in the control group (*p* = 0.15). Epiretinal membrane was present in 7.1% of the examined eyes in the post-COVID-19 group; a similar prevalence was reported for macular hole and vitreomacular traction. None of these was statistically more prevalent in this group compared to the healthy controls. Cotton wool spots were detected in 12.9% of the post-COVID-19 population compared to 0% in the control group (*p* = 0.09). No anterior ocular inflammation was observed in the anterior segment evaluation. Superficial ocular discomfort has been reported in several patients, 39 patients (55.7%) during the course of the disease and 28 patients (40%) described the persistence of ocular discomfort symptoms after healing by COVID-19. None of them showed conjunctivitis. Vitreous fibrillary degeneration with no signs of inflammation was detected in 10 eyes. To mention, one case of extra papillary focal retinal hemorrhage was observed in a 58-year-old male patient. His systemic history reported hypertension, previous history of cardiac ischemia, and recent coronary stent with double antiplatelet therapy (cardioaspirin and ticagrelor). Detailed baseline characteristics of the study population are reported in Table 1.

**Table 1:** Descriptive analysis of the study groups

Variable	Post COVID-19	Controls	*P*
Age (years)	53.7 ± 14 (CI 50.7–56.7)	44.7 ± 11.3 (CI 40–49.5)	0.006
Sex	M = 39/70 (55.7%) F = 31/70 (44.3%)	M = 8/22 (36.4%) F = 14/22 (63.6%)	0.061
BCVA (Snellen)	20/22	20/23	0.53
Systemic arterial hypertension	14/70 (20%)	3/22 (13.6%)	0.047
Diabetes	30/70 (42.8%)	2/22 (9.1%)	0.003
BMI	25.63 ± 4.6 (CI 22.89–27.24)	22.75 ± 3.2 (CI 20.9–24.1)	0.041
Chronic kidney disease	6/70 (8.6%)	1/22 (4.5%)	0.12
Cognitive impairment	5/70 (7.1%)	2/22 (9.1%)	0.78
Previous stroke	1/70 (1.4%)	1/22 (4.5%)	0.65
Autoimmune diseases	6/70 (8.6%)	2/22 (9.1%)	0.92
Vitreomacular traction	5/70 (7.1%)	2/22 (9.1%)	0.88
Epiretinal membrane	5/70 (7.1%)	1/22 (4.5%)	0.79
Macular hole	3/70 (4.3%)	0/22 (0%)	0.54
Cotton wool spots	9/70 (12.9%)	0/22	0.09
Previous vitreoretinal surgery	5/70 (7.1%)	1/22 (4.5%)	0.59
Myopia	12/70 (17.1%)	4/22 (18.2%)	0.93
Days since symptoms onset	60.3 ± 13.6		
Days since hospital discharge	36.1 ± 12.9		
Ocular symptoms during infection	41/70 (58.6%)		
Ocular symptoms post infection	30/70 (42.9%)		
Intensive care unit admission	9/70 (12.9%)		
Oxygen therapy	33/70 (45.8%)		
Non-invasive ventilation	11/70 (15.7%)		
Mechanical ventilation	4/70 (5.7%)		

**Fig. 1:**
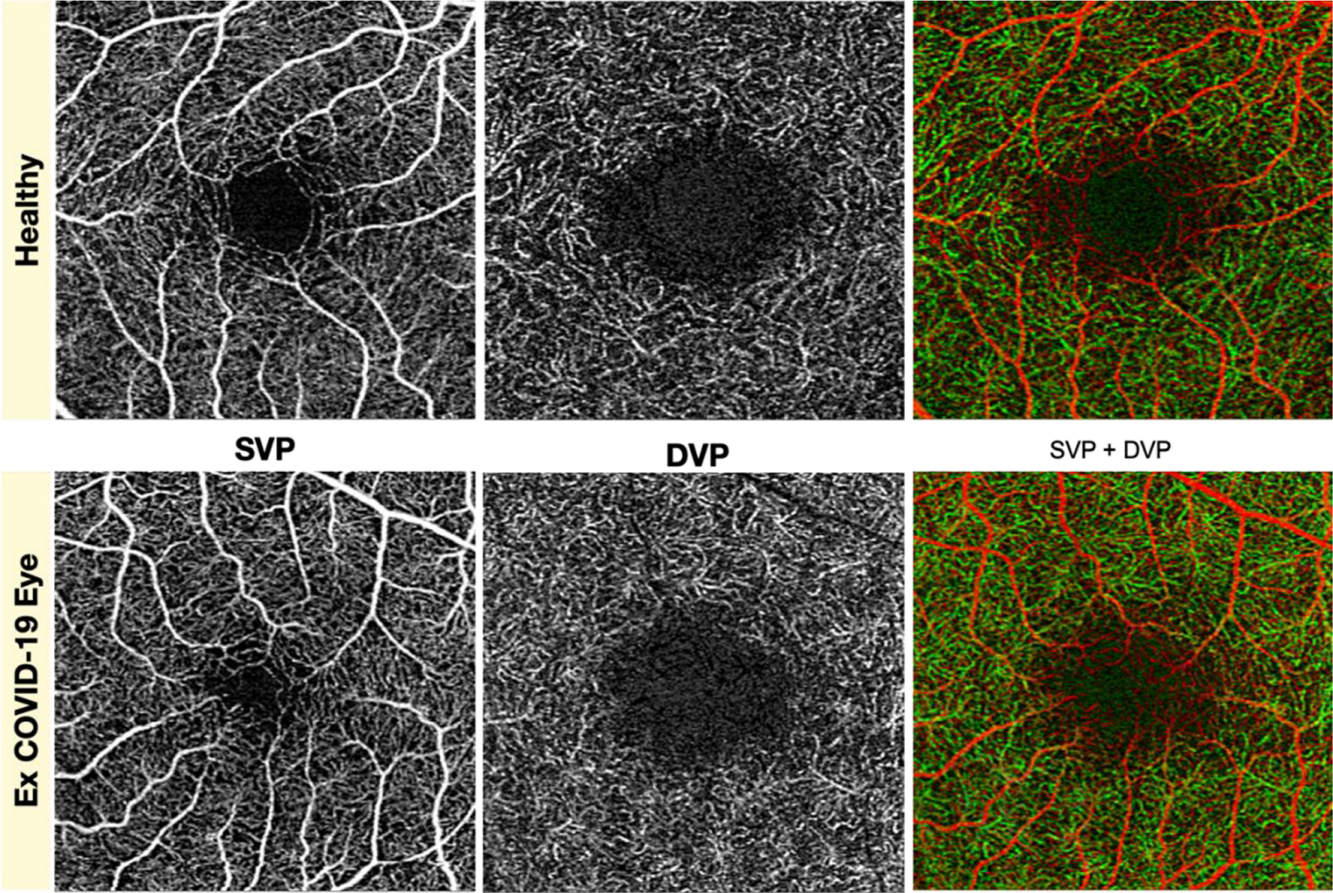
Superficial vascular plexus (SVP) and deep vascular plexus (DVP) taken individually and in association (SVP + DVP) in heathy and post-COVID-19 eye did not show differences at 1 month from hospital discharge

Mean SCP VD in the post-COVID-19 group at 3 × 3 mm and 6 × 6 mm was 21.20 ± 1.2% and 18.49 ± 1.18% respectively. Values of SCP VP extracted from the same acquisitions were 0.386 ± 0.02 for 3 × 3 mm and 0.459 ± 0.03 for 6 × 6 mm. With regard to DCP, mean vessel density in the post-COVID group was 21.82 ± 2.51% (3 × 3 acquisitions). Intraclass correlation coefficient revealed a good reliability of the measurements of subfoveal choroidal thickness and FAZ perimeter (respectively ICC = 0.884 *r* = 0.802 and ICC = 0.961, *r* = 0.929) (Fig. 1).

**Fig. 2:**
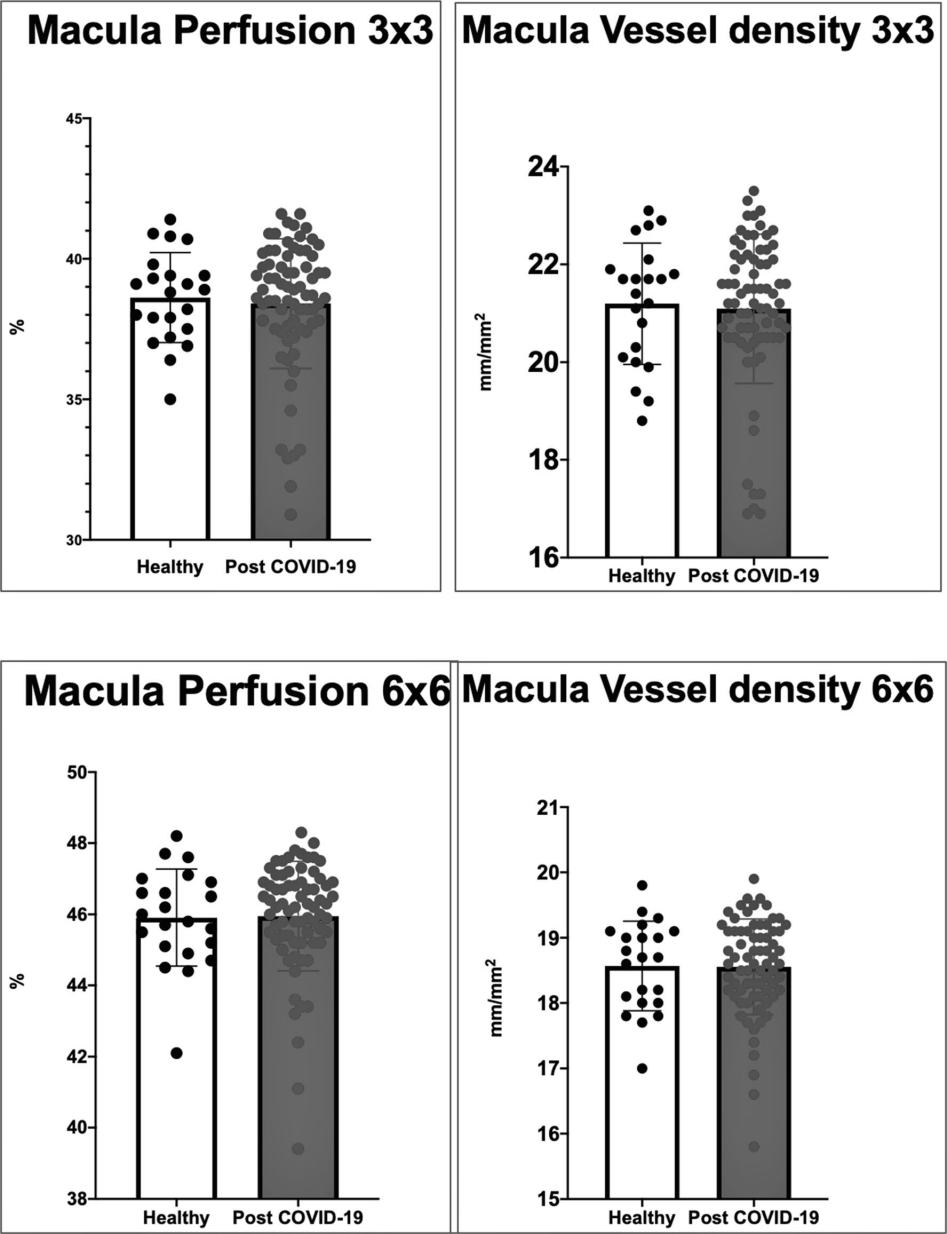
Histograms showing distributions of macular perfusion and macular vessel density in SCP between healthy and post-COVID-19 eyes in 3 × 3 mm and 6 × 6 mm posterior pole scan by OCT angiography. No significant differences in scores were found between post-COVID-19 patients and healthy controls

Results from the inferential analysis revealed no significant differences between the two groups in terms of density and perfusion of SCP (see Fig. 2) or DCP. The comparison was equally inconclusive for 3 × 3 mm and 6 × 6 mm acquisitions. A binary logistic regression was performed to exclude confounding factors from the analysis but this led to no change in the detected differences.

**Fig. 3:**
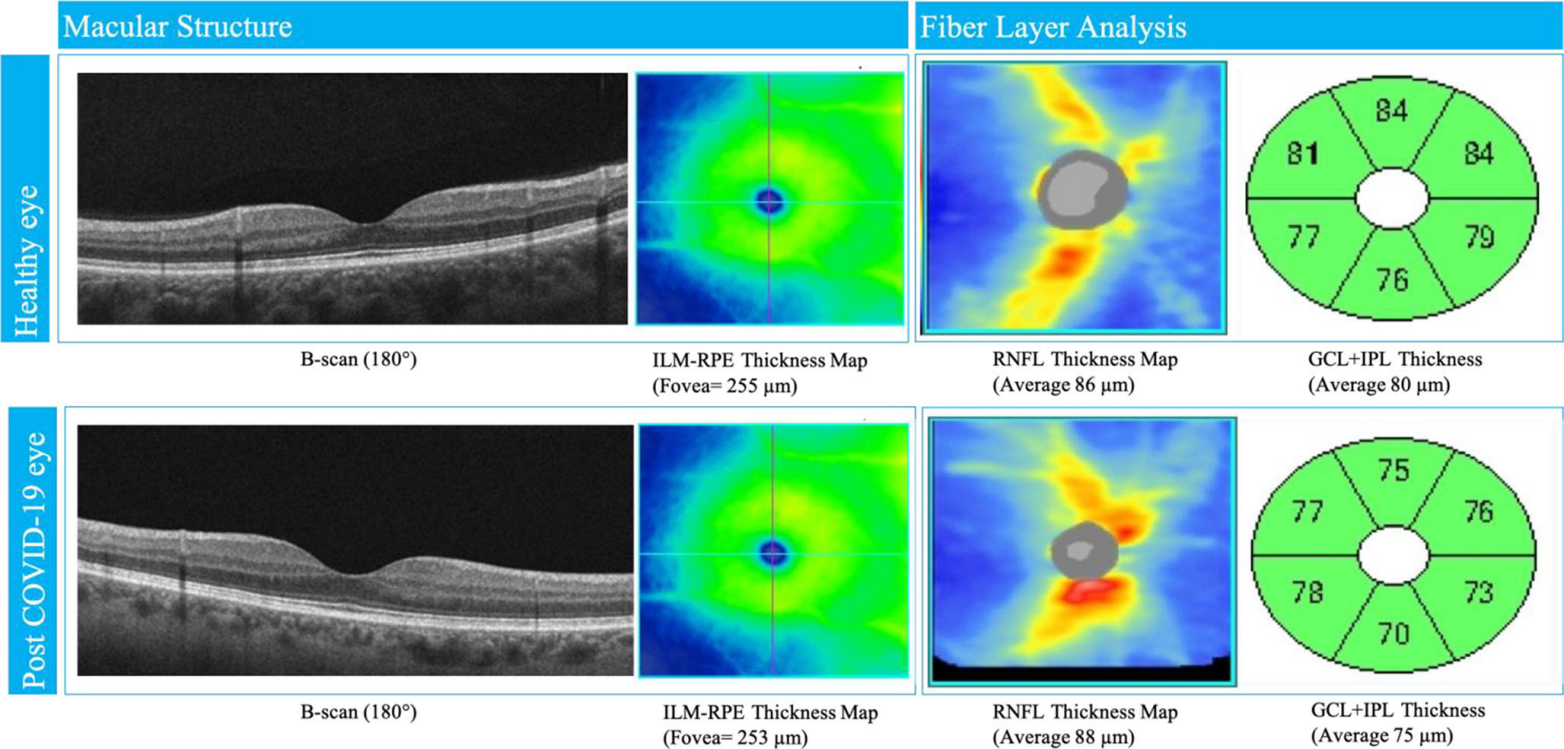
Structural OCT of macula and fiber layer in fovea and parapapillary region in healthy and post-COVID-19 eyes. Morphological parameters were similar in both cases. ILM, inner limiting membrane; RPE, retinal pigment epithelium; RNFL, retinal nerve fiber layer; GCL, ganglion cell layer; IPL, inner plexiform layer

Results from OCTA analysis in COVID-19 group reveal a mean FAZ area of 0.235 ± 0.11 mm^2^, with a FAZ perimeter of 2.04 ± 0.52 mm. Central foveal thickness and subfoveal choroidal thickness were 263.76 ± 25.68 μm and 304.83 ± 35.48 μm respectively. As concerns inner retinal layers, mean GCC thickness was 80.76 ± 9.60 μm while mean RNFL thickness was 94.62 ± 10.46 μm. Among the secondary outcome measures, neither OCTA nor structural OCT parameters (see Fig. 3) differed significantly between the two groups. Results from the regression analysis are summarized in Table 2.

**Table 2:** Inferential analysis of the differences between post-COVID and control groups as concerns primary and secondary outcomes. Statistical significance refers to the results of regression analysis

Outcome	Variable	Post COVID-19	Controls	*P*
Primary outcome	SCP 3 × 3 vessel density (%)	21.20 ± 1.2 (CI 20.68–21.72)	21.20 ± 1.4 (CI 20.89–21.50)	0.99
	SCP 6 × 6 vessel density (%)	18.49 ± 1.18 (CI 18.23–18.74)	18.57 ± 0.68 (CI 18.28–18.86)	0.76
	SCP 3 × 3 vessel perfusion	0.386 ± 0.02 (CI 0.381–0.391)	0.386 ± 0.02 ( CI 0.379–0.393)	0.96
	SCP 6 × 6 vessel perfusion	0.459 ± 0.03 (CI 0.453–0.466)	0.459 ± 0.01 ( CI 0.453–0.465)	0.96
	DCP 3 × 3 vessel density (%)	21.82 ± 2.51 (CI 19.75–24.10)	23.05 ± 3.44 (CI 21.23–25.76)	0.88
Secondary outcome	FAZ area (mm^2^)	0.235 ± 0.11 (CI 0.211–0.258)	0.235 ± 0.09 (CI 0.196–0.274)	0.98
	FAZ perimeter (mm)	2.04 ± 0.52 (CI 1.93–2.15)	2.06 ± 0.36 (1.91–2.21)	0.87
	Central foveal thickness (μm)	263.76 ± 25.68 (CI 258.24–269.28)	266.91 ± 18.34 (CI 259.25–274.57)	0.59
	Subfoveal choroidal thickness (μm)	304.83 ± 35.48 (CI 287.52–322.15)	312.55 ± 50.03 (CI 270.61–354.48)	0.70
	GCC average thickness (μm)	80.76 ± 9.60 (CI 78.69–82.82)	81.27 ± 4.81 (CI 79.26–83.28)	0.81
	RNFL average thickness (μm)	94.62 ± 10.46 (CI 92.36–96.89)	93.73 ± 8.12 (CI 90.33–97.12)	0.71

## Discussion

The results of our study suggest that there isn’t any relevant difference between early post-COVID-19 patients and general population in terms of VD in the SCP and DCP. VP of SCP, FAZ area, and FAZ perimeter were found to be equally similar between the two analyzed populations. In addition, none of the OCT-B scans revealed any structural modification in early post-COVID-19 patients. Our study detected a prevalence of 12.9% of cotton wool spots in post-COVID-19 population, a prevalence that didn’t happen to differ significantly from that of the control population according to regression analysis. To our knowledge, this is the first cohort study and the largest scale experimental evidence to address this matter in the early postinfective period of COVID-19 disease. A recent cross-sectional study from Abrishami et al. [28] evaluated 31 patients 2 weeks after recovery from COVID-19 and detected a statistically significant lower foveal and parafoveal VD both in SCP and DCP compared to a retrospective healthy cohort. In our opinion, this apparently conflicting result must be interpreted carefully in consideration of the higher prevalence of immune diseases, obesity, diabetes, and cardiovascular diseases in patients affected by moderate symptomatic forms of COVID-19 infection [29]. In fact, systemic conditions like the above mentioned are potentially associated to structural and functional vascular changes in the retina as extensively demonstrated by the scientific literature [30]. The lack of retrospective data and the small sample size precluding the possibility of stratification of the population for associated medical conditions impose a major limit to the reported finding. Landecho et al. [31] suggested how COVID-19 microangiopathy could serve as an in vivo biomarker of systemic vascular disease as a conclusion to the finding of a 22% of retinal microangiopathy at a mean of 43 days after COVID-19 first symptom. In favor of this theory, our study found a higher mean BMI in post-COVID-19 patients, displaying a trend to linear correlation with SCP perfusion in 6 × 6 mm acquisitions. Another important consideration is that our study analyzed a subgroup of patients afflicted by a moderate form of the disease (low prevalence of life-threatening complications and ICU admissions) and affected by a relatively low burden of aggravating systemic conditions compared to data in the literature for hospitalized COVID-19 patients [32]. The first occurrence of the two is coherent to the fact that 81% of patients with COVID-19 manifest a mild form of the disease [33]. Nevertheless, this could be one of the possible explanations for the absence of findings suggestive of macular microvascular impairment: this kind of damage could be an occurrence restricted to severely affected COVID-19 patients. Lastly, another possible explanation is the reversibility of any damage to macular capillary plexuses after the resolution of the acute phase of the infection. In fact, during the acute phase of the disease, retinal hemorrhages were found in 9%, cotton wools spots in 7%, dilated veins in 28%, and tortuous vessels in 13% of COVID-19 patients [34]. Moreover, accounting for covariates, mean vein diameter was positively associated with COVID-19 both in severe and non-severe cases compared to unexposed subjects, and it was negatively correlated with the time from symptoms onset and positively correlated with disease severity [34]. Marinho et al. also described four patients who presented subtle cotton wool spots and microhemorrhages along the retinal arcade, with no symptoms or signs of intraocular inflammation [35]. Another case of retinal papillophlebitis was described in a 40-year-old COVID-19 patient presenting with dilated and tortuous retinal vessels, disc edema, and retinal hemorrhages [36].

As to our experience, we similarly observed one case of extra papillary focal retinal hemorrhage. However, our patient had hypertension, previous history of cardiac ischemia, and a recent coronary stent and was under double antiplatelet therapy.

Our results offer the perspective of an absence of retinal macular involvement in the early post-infective phase of this concerning disease. What this paper has to offer to the scientific community is therefore a hint of a good news. Nevertheless, it should be considered that the impossibility to perform fluorescein angiography as part of the study evaluation due to the unjustified invasiveness of the exam provides some limitations to the study findings. 

 An additional limitation that should be considered is the small number of patients enrolled in the control group; nevertheless, according to the sample size calculation, it would have provided sufficient strength in order to detect changes in the between group comparisons. Another limiting feature was the younger age of patients in the control group. By the way, it is important to notice that this difference was merely statistical and characterized by a scarce clinical relevance (mean age of 54 in the post-COVID group versus mean age of 45 in control group).

Further studies will be needed to address this important subject of actuality and confirm or contrast our findings, also focusing on the subgroup of post-COVID-19 patient which is more susceptible to possible retinal microvascular sequelae.

## References

[1] WHO, W. (2020). WHO Director-General’s opening remarks at the media briefing on COVID-19.

[2] Li G, Fan Y, Lai Y, Han T, Li Z, Zhou P, Pan P, Wang W, Hu D, Liu X, Zhang Q, Wu J (2020). Coronavirus infections and immune responses. J Med Virol.

[3] Menter T, Haslbauer JD, & Nienhold R. (2020). Post-mortem examination of COVID-19 patients reveals diffuse alveolar damage with severe capillary congestion and variegated findings of lungs and other organs suggesting vascular dysfunction. 77(2), 198–209. 10.1111/his.14134 PMC7496150 32364264

[4] Marchetti M (2020). COVID-19-driven endothelial damage: complement, HIF-1, and ABL2 are potential pathways of damage and targets for cure. Ann Hematol.

[5] Saba, L., & Sverzellati, N. (2020). Is COVID evolution due to occurrence of pulmonary vascular thrombosis? Journal of Thoracic Imaging. 10.1097/RTI.0000000000000530 10.1097/RTI.0000000000000530 PMC7253049 32349055

[6] Chen L, Li X, Chen M, Feng Y, Xiong C (2020). The ACE2 expression in human heart indicates new potential mechanism of heart injury among patients infected with SARS-CoV-2. Cardiovasc Res.

[7] Zhu P, Verma A, Prasad T, Li Q (2020). Expression and function of Mas-related G protein-coupled receptor D and its ligand alamandine in retina. Mol Neurobiol.

[8] Casagrande M, Fitzek A, Püschel K, Aleshcheva G, Schultheiss H-P, Berneking L, Spitzer MS, Schultheiss M (2020). Detection of SARS-CoV-2 in human retinal biopsies of deceased COVID-19 patients. Ocul Immunol Inflamm.

[9] Donati, S., Maresca, A. M., Cattaneo, J., Grossi, A., Mazzola, M., Caprani, S. M., Premoli, L., Docchio, F., Rizzoni, D., Guasti, L., & Azzolini, C. (2019). Optical coherence tomography angiography and arterial hypertension: a role in identifying subclinical microvascular damage? European Journal of Ophthalmology, 1120672119880390. 10.1177/1120672119880390 10.1177/1120672119880390 31617414

[10] Kashani AH, Chen C-L, Gahm JK, Zheng F, Richter GM, Rosenfeld PJ, Shi Y, Wang RK (2017). Optical coherence tomography angiography: a comprehensive review of current methods and clinical applications. Prog Retin Eye Res.

[11] Gemelli Against COVID-19 Post-Acute Care Study Group (2020). Post-COVID-19 global health strategies: the need for an interdisciplinary approach. Aging Clin Exp Res.

[12] Al-Sheikh M, Phasukkijwatana N, Dolz-Marco R, Rahimi M, Iafe NA, Freund KB, Sadda SR, Sarraf D (2017). Quantitative OCT angiography of the retinal microvasculature and the choriocapillaris in myopic eyes. Invest Ophthalmol Vis Sci.

[13] Milani P, Montesano G, Rossetti L, Bergamini F, Pece A (2018). Vessel density, retinal thickness, and choriocapillaris vascular flow in myopic eyes on OCT angiography. Graefe’s Archive for Clinical and Experimental Ophthalmology = Albrecht Von Graefes Archiv Fur Klinische Und Experimentelle Ophthalmologie.

[14] Daniels AB, Froehler MT, Nunnally AH, Pierce JM, Bozic I, Stone CA, Santapuram PR, Tao YK, Boyd KL, Himmel LE, Chen S-C, Du L, Friedman DL, Richmond A (2019). Rabbit model of intra-arterial chemotherapy toxicity demonstrates retinopathy and vasculopathy related to drug and dose, not procedure or approach. Invest Ophthalmol Vis Sci.

[15] Minnella AM, Barbano L, & Verrecchia E. (2019). Macular impairment in Fabry disease: a morpho-functional assessment by swept-source OCT angiography and focal electroretinography. 60(7):2667-2675. 10.1167/iovs.18-26052 31242288

[16] Rosenfeld PJ, Durbin MK, Roisman L, Zheng F, Miller A, Robbins G, Schaal KB, Gregori G (2016). ZEISS Angioplex^TM^ spectral domain optical coherence tomography angiography: technical aspects. Dev Ophthalmol.

[17] Lu Y, Wang JC, Zeng R, Katz R, Vavvas DG, Miller JW, Miller JB (2019) Quantitative comparison of microvascular metrics on three optical coherence tomography angiography devices in chorioretinal disease. Clinical Ophthalmology (Auckland, N.Z.), 13, 2063–2069. 10.2147/OPTH.S215322 10.2147/OPTH.S215322 PMC6816077 31749603

[18] Kashani AH, Zhang Y, Capone A, Drenser KA, Puliafito C, Moshfeghi AA, Williams GA, Trese MT (2016). Impaired retinal perfusion resulting from vitreoretinal traction: a mechanism of retinal vascular insufficiency. Ophthalmic Surgery, Lasers & Imaging Retina.

[19] Chen H, Chi W, Cai X, Deng Y, Jiang X, Wei Y, Zhang S (2019). Macular microvasculature features before and after vitrectomy in idiopathic macular epiretinal membrane: an OCT angiography analysis. Eye.

[20] Wilczyński T, Heinke A, Niedzielska-Krycia A, Jorg D, Michalska-Małecka K (2019). Optical coherence tomography angiography features in patients with idiopathic full-thickness macular hole, before and after surgical treatment. Clin Interv Aging.

[21] Fan H, Chen H-Y, Ma H-J, Chang Z, Yin H-Q, Ng DS-C, Cheung CY, Hu S, Xiang X, Tang S-B, Li S-N (2017). Reduced macular vascular density in myopic eyes. Chin Med J.

[22] Romano MR, Cennamo G, Schiemer S, Rossi C, Sparnelli F, Cennamo G (2017). Deep and superficial OCT angiography changes after macular peeling: idiopathic vs diabetic epiretinal membranes. Graefe’s Archive for Clinical and Experimental Ophthalmology = Albrecht Von Graefes Archiv Fur Klinische Und Experimentelle Ophthalmologie.

[23] Vujosevic S, Toma C, Villani E, Gatti V, Brambilla M, Muraca A, Ponziani MC, Aimaretti G, Nuzzo A, Nucci P, De Cilla’, S. (2019). Early detection of microvascular changes in patients with diabetes mellitus without and with diabetic retinopathy: comparison between different swept-source OCT-A instruments. J Diabetes Res.

[24] Takayama K, Kaneko H, Ito Y, Kataoka K, Iwase T, Yasuma T, Matsuura T, Tsunekawa T, Shimizu H, Suzumura A, Ra E, Akahori T, Terasaki H (2018). Novel classification of early-stage systemic hypertensive changes in human retina based on OCTA measurement of choriocapillaris. Sci Rep.

[25] Jiang H, Wei Y, Shi Y, Wright CB, Sun X, Gregori G, Zheng F, Vanner EA, Lam BL, Rundek T, Wang J (2018). Altered macular microvasculature in mild cognitive impairment and Alzheimer disease. Journal of Neuro-Ophthalmology: The Official Journal of the North American Neuro-Ophthalmology Society.

[26] Peng C, Kwapong WR, Xu S, Muse FM, Yan J, Qu M, Cao Y, Miao H, Zhen Z, Wu B, Han Z (2020). Structural and microvascular changes in the macular are associated with severity of white matter lesions. Front Neurol.

[27] Vadalà M, Castellucci M, Guarrasi G, Terrasi M, La Blasca T, Mulè G (2019). Retinal and choroidal vasculature changes associated with chronic kidney disease. Graefe’s Archive for Clinical and Experimental Ophthalmology = Albrecht Von Graefes Archiv Fur Klinische Und Experimentelle Ophthalmologie.

[28] Abrishami M, Emamverdian Z, Shoeibi N, Omidtabrizi A, Daneshvar R, Rezvani TS, Saeedian N, Eslami S, Mazloumi M, Sadda S, Sarraf D (2020) Optical coherence tomography angiography analysis of the retina in patients recovered from COVID-19: a case-control study. Canadian Journal of Ophthalmology Journal Canadien D’ophtalmologie. 10.1016/j.jcjo.2020.11.006 10.1016/j.jcjo.2020.11.006 PMC7666612 33249111

[29] Vavvas, D. G., Sarraf, D., Sadda, S. R., Eliott, D., Ehlers, J. P., Waheed, N. K., Morizane, Y., Sakamoto, T., Tsilimbaris, M., & Miller, J. B. (2020). Concerns about the interpretation of OCT and fundus findings in COVID-19 patients in recent Lancet publication. Eye (London, England). 10.1038/s41433-020-1084-9 10.1038/s41433-020-1084-9 PMC7347265 32647303

[30] Flammer J, Konieczka K, Bruno RM, Virdis A, Flammer AJ, Taddei S (2013). The eye and the heart. Eur Heart J.

[31] Landecho MF, Yuste JR, Gándara E, Sunsundegui P, Quiroga J, Alcaide AB, García-Layana A (2020) COVID-19 retinal microangiopathy as an in vivo biomarker of systemic vascular disease? J Intern Med. 10.1111/joim.13156 10.1111/joim.13156 32729633

[32] Mazzaccaro D, Giacomazzi F, Giannetta M, Varriale A, Scaramuzzo R, Modafferi A, Malacrida G, Righini P, Marrocco-Trischitta MM, Nano G (2020). Non-overt coagulopathy in non-ICU patients with mild to moderate COVID-19 pneumonia. J Clin Med.

[33] Wu Z, McGoogan JM (2020). Characteristics of and important lessons from the coronavirus disease 2019 (COVID-19) outbreak in China: summary of a report of 72 314 cases from the Chinese Center for Disease Control and Prevention. JAMA.

[34] Invernizzi A, Torre A, Parrulli S, Zicarelli F, Schiuma M, Colombo V, Giacomelli A, Cigada M, Milazzo L, Ridolfo A, Faggion I, Cordier L, Oldani M, Marini S, Villa P, Rizzardini G, Galli M, Antinori S, Staurenghi G, Meroni L (2020) Retinal findings in patients with COVID-19: results from the SERPICO-19 study. EClinicalMedicine 100550. 10.1016/j.eclinm.2020.100550 10.1016/j.eclinm.2020.100550 PMC7502280 32984785

[35] Marinho PM, Marcos AAA, Romano AC, Nascimento H, Belfort R (2020) Retinal findings in patients with COVID-19. Lancet (London, England), 395(10237), 1610. 10.1016/S0140-6736(20)31014-X 10.1016/S0140-6736(20)31014-X PMC7217650 32405105

[36] Insausti-García, A., Reche-Sainz, J. A., Ruiz-Arranz, C., López Vázquez, Á., & Ferro-Osuna, M. (2020). Papillophlebitis in a COVID-19 patient: inflammation and hypercoagulable state. European Journal of Ophthalmology, 1120672120947591. 10.1177/1120672120947591 10.1177/1120672120947591 PMC7399568 32735134

